# A systematic review of the evidence for single stage and two stage revision of infected knee replacement

**DOI:** 10.1186/1471-2474-14-222

**Published:** 2013-07-29

**Authors:** James PM Masters, Nicholas A Smith, Pedro Foguet, Mike Reed, Helen Parsons, Andrew P Sprowson

**Affiliations:** 1 University of Warwick, Clinical Sciences Buildings, University Hospitals Coventry and Warwickshire, Coventry CV2 2DX, UK; 2 Department of Trauma and Orthopaedics, University Hospitals Coventry and Warwickshire, Coventry CV2 2DX, UK; 3 Department of Trauma and Orthopaedics, University Hospitals Coventry and Warwickshire, Coventry CV2 2DX, UK; 4 Division of Health Sciences, Warwick Medical School, University of Warwick, Coventry CV4 7AL, UK

**Keywords:** Infection, Knee replacement, One stage, Two-stage, Arthroplasty, Revision

## Abstract

**Background:**

Periprosthetic infection about the knee is a devastating complication that may affect between 1% and 5% of knee replacement. With over 79 000 knee replacements being implanted each year in the UK, periprosthetic infection (PJI) is set to become an important burden of disease and cost to the healthcare economy. One of the important controversies in treatment of PJI is whether a single stage revision operation is superior to a two-stage procedure. This study sought to systematically evaluate the published evidence to determine which technique had lowest reinfection rates.

**Methods:**

A systematic review of the literature was undertaken using the MEDLINE and EMBASE databases with the aim to identify existing studies that present the outcomes of each surgical technique. Reinfection rate was the primary outcome measure. Studies of specific subsets of patients such as resistant organisms were excluded.

**Results:**

63 studies were identified that met the inclusion criteria. The majority of which (58) were reports of two-stage revision. Reinfection rated varied between 0% and 41% in two-stage studies, and 0% and 11% in single stage studies. No clinical trials were identified and the majority of studies were observational studies.

**Conclusions:**

Evidence for both one-stage and two-stage revision is largely of low quality. The evidence basis for two-stage revision is significantly larger, and further work into direct comparison between the two techniques should be undertaken as a priority.

## Background

Knee replacement is a widely performed procedure for the management of knee arthritis. According to the 9th National Joint Registry report 79,516 knee replacements were undertaken in the UK in 2011 [1].

Surgical site infection (SSI) is estimated to complicate around 1% [2] of knee replacement and is considered amongst the most devastating complications that can affect this procedure. Based upon high quality surveillance in national programs the true rate appears to be between 3.3% [3] and 4.19% [4].

Postoperative SSI is classified by the health protection agency on the basis of depth of infection. Superficial infections are limited to the incision and superficial tissues. Deep infections involve the fascial layers and may occur up to one year post operatively where an implant is in place [5] and influences surgical management strategy [6].

Non-operative management with antibiotics is often reserved for patients unable to undergo surgery [7] and may have additional associated complications of antibiotic related organ damage.

Certain prosthesis retaining strategies may be used such as arthroscopic debridement [8], open debridement with removal of the polyethylene spacer [9] and surgical debridement, antibiotics and implant retention (DAIR) [10]. However the efficacy is somewhat controversial [11] with a poorer rate of success at eradicating the infection than more radical strategies which involve implant removal followed by implantation of a new prosthetic joint.

The most widespread technique used to perform this knee revision surgery is the two-stage method. This involves an index procedure where thorough irrigation and debridement of infected tissue is performed and the infected prosthesis is removed from the joint. In its place is usually a cement ‘spacer’. The spacer is a block of cement, often containing antibiotics, that is placed in the remaining knee space to maintain the muscle and soft tissue tension. More recently articulating spacers have been used which allow a degree of movement at the knee joint. The spacer is left within the knee joint for a period of between 6 and 8 weeks during which the patient receives parenteral antibiotics [12]. After the 6-week interval a second procedure is carried out and a new definitive prosthesis is inserted. This procedure is currently considered gold standard. The disadvantages of two-stage surgery, include the need for two operations and a potentially lengthy inpatient stay. The interval between index and second procedure can impair mobility and soft tissue contractures may develop. Mobile articulating spacers have been developed to help reduce this problem. In addition there is a significant financial burden associated with this treatment protocol [13].

An alternative to the two-stage revision is a condensed ‘one-stage’ procedure that is growing in popularity [14]. This involves a single procedure in which the definitive revision prosthesis is inserted during the index operation after removal of the infected knee replacement and an extensive debridement of all infected tissue. The potential benefits of one-stage revision are reduced morbidity, improved functional outcome as well as economic benefits [14-16].

This paper seeks to systematically review the evidence for the use of both one-stage and two-stage revision for infected knee replacement.

## Methods

A systematic review of the literature was undertaken according to the methods described in the *Cochrane Handbook for Systematic Review of Interventions*[17].

### Research question

#### Participants

Any patient with infected knee replacement as defined by the study reviewed.

#### Intervention

Revision knee surgery where all infected tissue and components are removed and a new definitive implant inserted in a single procedure. Hereafter referred to as ‘One-stage’.

#### Comparator

Revision knee surgery where infected tissue and components are removed and definitive implants are inserted in a separate surgical session. Hereafter referred to as ‘Two-stage’.

#### Primary outcome

Reinfection rate

#### Secondary outcomes

Functional scores at last follow up

Range of movement at last follow up

### Exclusion criteria

Studies were excluded where patients were selected on the basis of having a specific subset of periprosthetic joint infection e.g. antibiotic resistant organisms.

Studies were limited to those published in the English language and humans. If studies presented a mixed group of treatments e.g. arthrodesis, one-stage and debridement, they were excluded if the treatment specific outcome was not presented. Studies reporting data on both hip and knee revision were only included if data for the knee was available for independent analysis. Case studies, abstracts, reviews and unpublished data were not included. Studies with less than one-year minimum follow up and fewer than 5 patients were excluded. Our scoping review of the literature identified that very few studies identified whether the revision surgery was preceded by previous attempts at revision. It is therefore not possible, in the majority of cases, to identify whether the cases are recurrent infection, index infection or a mixture.

### Statistics

Due to the anticipated clinical heterogeneity of the studies no summative statistics were performed. The outcomes of all studies are presented in independent forest plots, with a point estimate for re-infection rate at last follow-up. 95% confidence intervals were calculated using a normal approximation interval formula for binomial data.

### Search strategy

The Embase and Medline databases were searched on the 15th December 2012 using the Ovid interface. The search strategy was modified from that used by Beswick and colleagues to identify similar papers regarding treatment of infected hip replacement [18]. See Additional file 1 for search terms used.

**Figure 1:**
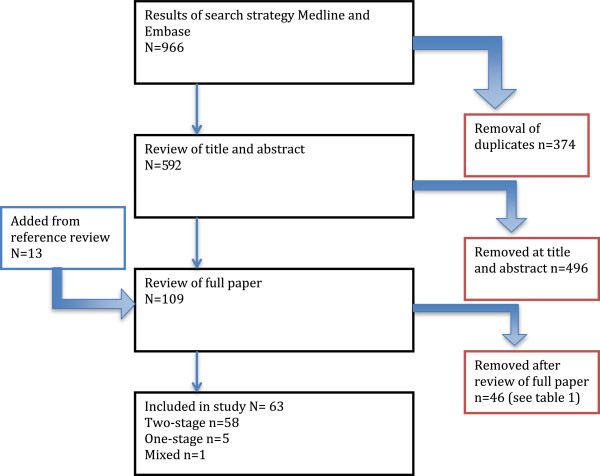
Study selection flowchart.

References were transferred into Endnote referencing software and duplicates were discarded. Firstly titles and abstracts were reviewed for relevance according to the research question. The remaining studies were analysed in their entirety. References of full texts were also reviewed to identify any other potentially relevant study. The acquisition of articles is summarised in a flow chart (Figure 1). Where there was discrepancy an agreement was reached by consensus.

Data were extracted by two reviewers (JM and NS), with discrepancies resolved by discussion. Authors were not contacted.

## Results and discussion

The search strategy yielded 644 individual papers. After review of titles, abstracts and language, 548 papers were excluded. The full text of 96 papers were reviewed along with an additional 13 studies identified from the review of references. There were 46 studies excluded for reasons detailed in Additional file 2. This left 58 studies of two-stage revisions (Table 1) and 5 studies of one-stage revisions (Table 2). Studies containing 45 patients or more were presented in the forest plot of two-stage studies. All studies are included in the results and discussion. The flow of this process is demonstrated in Figure 1.

**Table 1:** Summary of two-stage studies

**Name of study**	**Number of knees**	**Reinfection number (percentage)**	**Range of movement (degrees)**	**Follow up**	**Function scoring**	**Average age (years) and range**	**Gender distribution**
Fehring 2000	55 (30 articulating/25 static) all with antibiotics	4 (7%)	98 in static spacers 105 in articulating spacers	Static spacers 36 months (range, 24–72 months). Articulating spacers was 27 months (range, 24–36 months)	Static spacer- 83 points (HSS) Articulating spacer 84 points (HSS)	Not reported	Not reported
Ferrari 2011	50	4 patients 8%	Not reported	Followed up for a minimum of 24 months maximum 126	Mean IKSS clinical was 35.38 (clinical) & 37.96 (function) on presentation; it improved to a mean of 75.38 (clinical) & 80.58 (function) at the final review. Mean WOMAC (function and pain scores) was respectively 17.38 and 60.67 on presentation, it improved to a mean of 8.67 and 31.04 at final review	Not reported	Not reported
Freeman 2007	76 procedures in 74 patients, Static spacers were used in 28 procedures, and articulating spacers were used in 48 procedures	Articulating group was 5.3% (4 of 76 knees) compared to 7.9% (3 of 38 knees) in the static group.	Not reported	71.2 months (range, 24–196 months)	No significant difference between articulating and static spacers in KSS pain and functional scores, although the functional scores were higher in articulating group.	67 (range, 41-87 years)	38 were men and 36 were women
Goldman 1996	64	6 patients (9%)	Average 94 (30–120)	Average 7.5 years (2–17 years)	Mean HSS score 78 points	Average 67 (37–89)	21 males 39 females
Gooding 2011	110 patients 115 knees	14 knees (13%)	Postoperative knee flexion of 93.2 (range, 30 –140) and preoperative knee flexion of 86.2 (range, 15 –140)	Minimum 5 years	We observed an improvement in the mean postoperative WOMAC function, WOMAC pain, and WOMAC global scores. An improvement was also noted in the Oxford and the SF-12 (mental) scores and the satisfaction scores were recorded at last follow-up. The mean postoperative UCLA score at final follow-up was 4.1. WOMAC function (p = 0.001), WOMAC pain (p = 0.02), and WOMAC global (p = 0.002) scores as well as the Oxford (p = 0.0003) and the SF-12 (mental) (p = 0.008) scores all improved at last follow-up	68 years (range, 35–86 years)	(60 male and 50 female)
Haddad 2000	45	4 failures (9%)	Mean flexion at final follow-up 94.5 (20–135)	Mean follow-up 48 months (20–112)	Mean HSS score 71.4	69 years (26–83)	26 women 19 men
Haleem 2004	94 patients and 96 knees	9 knees (9%)	ROM at last follow-up had a median of 90° (range 30°–120°).	Median follow-up was 7.2 years (range, 2.5–13.2 years)	Preoperative KSS pain scores improved (p 0.001) from a median of 49 points (range, 4–85 points) to a median of 89 points (range, 35–97 points) postoperatively Preoperative functional scores improved (p 0.001) from a median of 5 points (range, 0–80 points) to a median of 50 points (range, 0–100 points) postoperatively.	69 years (range, 37–89 years)	50 men and 44 women
Hart 2006	48	6 patients (12%)	Mean fixed-flexion deformity of 1° (0° to 15°). Five patients had a fixed-flexion deformity of more than 10°. The mean maximum flexion was 92° (30° to 120°)	Average 48.5 months (26–85)	Not reported	Mean age 68.2 (37.2-81.3)	28 men 20 women
Hirakawa 1998	55 knees 54 patients	14 knees developed recurrent deep infection at an average of 50.8 weeks (range, 12–144)	Average knee flexion was 92° (range, 72°- 128°) before infection and 83° (range, 54°-130°) after reimplantation	61.9 months (range, 28–146 months).	Average HSS knee score was 85.3 points (range, 69–100) before infection and 78.6 points (range, 52–98) after reimplantation. NB In successful only	67 years (range, 41-83 years)	29 women and 25 men
Johnson 2012	111 patients 115 knees (Dynamic 34 Static 81)	Six patients in the dynamic spacer cohort (17%; 95% CI, 8%–34%) and 14 patients in the static spacer cohort (17%; 95% CI, 10%–27%)	Postoperative Knee Society objective scores and ROMs improved to 83 points (range, 48–99 points; 95% CI, 79–87 points) and 99″ (range, 60″–120‵; 95% CI, 92‶–104‶), respectively, for the dynamic spacer group and 84 points (range, 48–100 points; 95% CI, 81–87 points) and 95′ (range, 30″–130‶; 95% CI, 90″–101‶),	(Dynamic spacer group: mean, 27 months; range, 12–72 months; static spacer group: mean, 66 months; range, 12–121 months)	See rom box	Dynamic 62 (59–65) Static 61 (58–64)	Not Reported
Kalore 2012	53	15/53(28%) overall- (5/15 AOC; 2/16 NFC; 8/22 SMC)	95.7, 98.3, and 93.8 for SMC, NFC, and AOC spacers, respectively	Mean 39 months (12–105)	Not reported	Mean 64	38 men 15 women
Kurd 2010	96	26 patients (27%) (14 reinfected with same micro-organism)	Na	(Mean, 35 months; range, 24–90 months	Not reported	67 years (range, 17–88 years)	46 women and 56 men (6 subsequently lost to f/u)
Lonner 2001	53	9 (17%)	Not reported	56 months (24–144)	Not reported	Not reported	not reported
Mahmud 2012	253 knees	16 failures (7%)	Not reported	Median 48/12 (12–276)	The preoperative WOMAC score, and The Knee Society Clinical Rating scores were 48 (± 21) and 64 (± 31), respectively. The postoperative WOMAC and The Knee Society Clinical Rating scores were 60 (± 21) and 129 (± 41), respectively. The difference between the pre- and postoperative WOMAC and The Knee Society Clinical Rating scores were 12 and 65, respectively.	Mean age of 70 ± 10 years	104 were men and 135 women
Meek 2004	54 (47)	2 (4%)	N = 47 RoM preop 78.2 (SD 29.4); RoM postop 87.1 (14.7)	Average follow up 41 months	SF12 Mental 53.7 (11.9); SF12 Physical 41.2 (13.4); Oxford 67.3 (24.3); WOMAC-function 68.9 (21.9)-pain 77.1 (25.2)-stiffness 70.2 (24.2)	Not reported	27 females and 20 males
Mont 2000	69 two stage (group I-35, group II-34)	Group I-5/35 (14%; Group II 1/34 (3%). Total reinfection rate 6/69 (8.7%)		Group I 68 months (36–114); group II 58 months (36–91)	KSS (infection free knee only) 86 (80–95) in group I; 88 (64–98) in group II	Group I 64 (46–80); Group II 69 (56–82)	M:F group I 17:18 group II 16:18
Mortazavi 2011	117	33 failures (28%)	Not reported	Mean followup was 3.8 years (range, 2–9.4 years)	No functional outcomes reported	67.5 years (range, 37–88 years)	55 (47%) were female
Van Thiel 2011	58	7 failures (12%)	The mean extension before placement of the spacer was 3.2 (range, 0 –30) and a mean of 2.0 at final followup (range, 0 –10); the mean pretreatment flexion of 90.6 (range, 10 –125) improved to a mean of 101.3 (range, 0 –130) at final followup	(mean, 35 months; range, 24–51 months)	Mean pretreatment Knee Society score of 53 (range, 10–100) improved to a mean of 79 (range, 37–100) at most recent followup	66 years (range, 42–91 years).	29 women and 31 men
Westrich 2010	72 patients (75 knees)	7 knees (9.3%)	Not reported	52.4 months (range, 24–108 months)	Mean Knee Society knee score improved from 65.1 preoperatively to 90.1 at last follow-up, and the mean Knee Society functional score improved from 29.4 preoperatively to 90 at last follow-up.	65.5 (range, 39–86)	37 men (51.4%) and 35 women (48.6%)
Hofman 2005	50	6 patients (12%)	Pre reimplantation 6–91; Post implantation 4-104	73 months (24–150)	Average modified HSS after revision 89pts(70–100)	67 yrs (38–92)	25 men 25 women

**Table 2:** Summary of one stage studies

**Name/Year**	**Number**	**Reinfection rate**	**Range of movement**	**Follow-up**	**Other functional outcome**	**Gender**	**Age**
Freeman 1985	8	0	Not reported	12-40 months	Not reported	6 women 2 men	47 to 78 years
Goksan 1992	18	11%	Mean 87 degree flexion	Mean 5 years	Not reported	12 women 6 men	Mean age 61.4 (42–74)
Singer 2012	63	3 (5%)	The mean degree of flexion 2 years after surgery was 104 ± 11	24 months (mean, 36 months; range, 24–70 months)	Mean Knee Society knee score 24 months after surgery was 72 points (range, 20–98 points), the Knee Society function score was 71 points (range, 10–100 points), and the Oxford-12 knee score was 27 points (range, 13–44 points).	32 women and 31 men	70.7 ± 10.5 years (range, 31–89 years).
Buechel 2004 (II)	21	2 (9.1%)	Not reported	10.2 year (1.4-19.6)	Average final post op knee score 79.5 (35–94)	13 female 9 male	Mean age 70.6 (58-86)

No clinical trials were identified in the search; all studies found in this search were observational studies of patients who had undergone either treatment protocol. As no randomised studies were identified, no meta-analytical techniques were used due to the great clinical and statistical heterogeneity expected amongst the studies.

### One-stage

Four studies (Table 2) described the outcomes of patients exclusively undergoing one-stage revision. The re-infection rates reported varied from 0% [19], 5% [20], 9.1% [21] and 11% [22].

The largest series of one-stage revisions was reported by Singer, which details the results of 63 infected knee arthroplasties. In patients with infected primary and unicompartmental knee arthroplasties (n = 43) there were no recurrences of infection within the 24 month follow up. The recurrences of infection were found in patients who had undergone previous revision surgery.

An earlier study by Buechel and colleagues found similar results; this case series of 21 patients demonstrated a re-infection rate of 9.1%.

In terms of functional outcomes, Singer et al. reported a mean Knee Society Score of 72 points after 24 months and a mean reported range of movement of 104°. Buechel et al. had a similar mean final postoperative knee score of 79.5 (range 35–94).

Two studies from the UK report a much earlier experience with the one-stage technique [19,22] and present re-infection rates of 11% and 0%. No validated functional outcomes were reported, however the reports describe that all patients were walking and had flexion of greater than 90 and 70 degrees respectively.

One study contained cohorts of patients who had undergone one-stage and two-stage revision [23]. This study from 1987 had one patient who underwent a one-stage procedure as their index revision procedure; this patient successfully cleared the infection. One stage revision was also used in two patients who failed a prosthesis retaining debridement also successfully cleared the infection.

Two-stage revision was used as a primary revision technique in 9 patients with 1 failure. An additional two patients underwent two-stage revision having failed to clear infection after debridement. All patients in the two-stage group were identified as chronic infections. The authors report that acute infections occurring in less than two weeks were treated preferentially by component retention and debridement. This study has a single stage revision failure rate of 0/3 (0%) and a two stage failure rate of 1/11 (9%). These two results are split respectively across the two forest plots.

**Figure 2:**
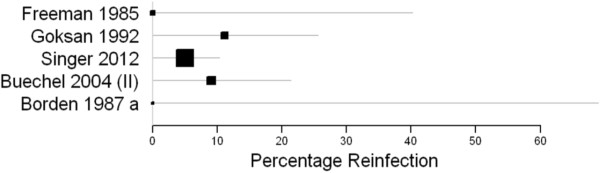
**Forest plot-one stage revision.** X-axis is point estimate of reinfection at last follow up, which is variable, presented with 95% confidence intervals. ‘Borden 1987 a’ represents the results of the single stage revision presented in this paper.

The re-infection rates with 95 percent confidence intervals show a very wide range (Figure 2). This highlights small study numbers.

### Two-stage

**Figure 3:**
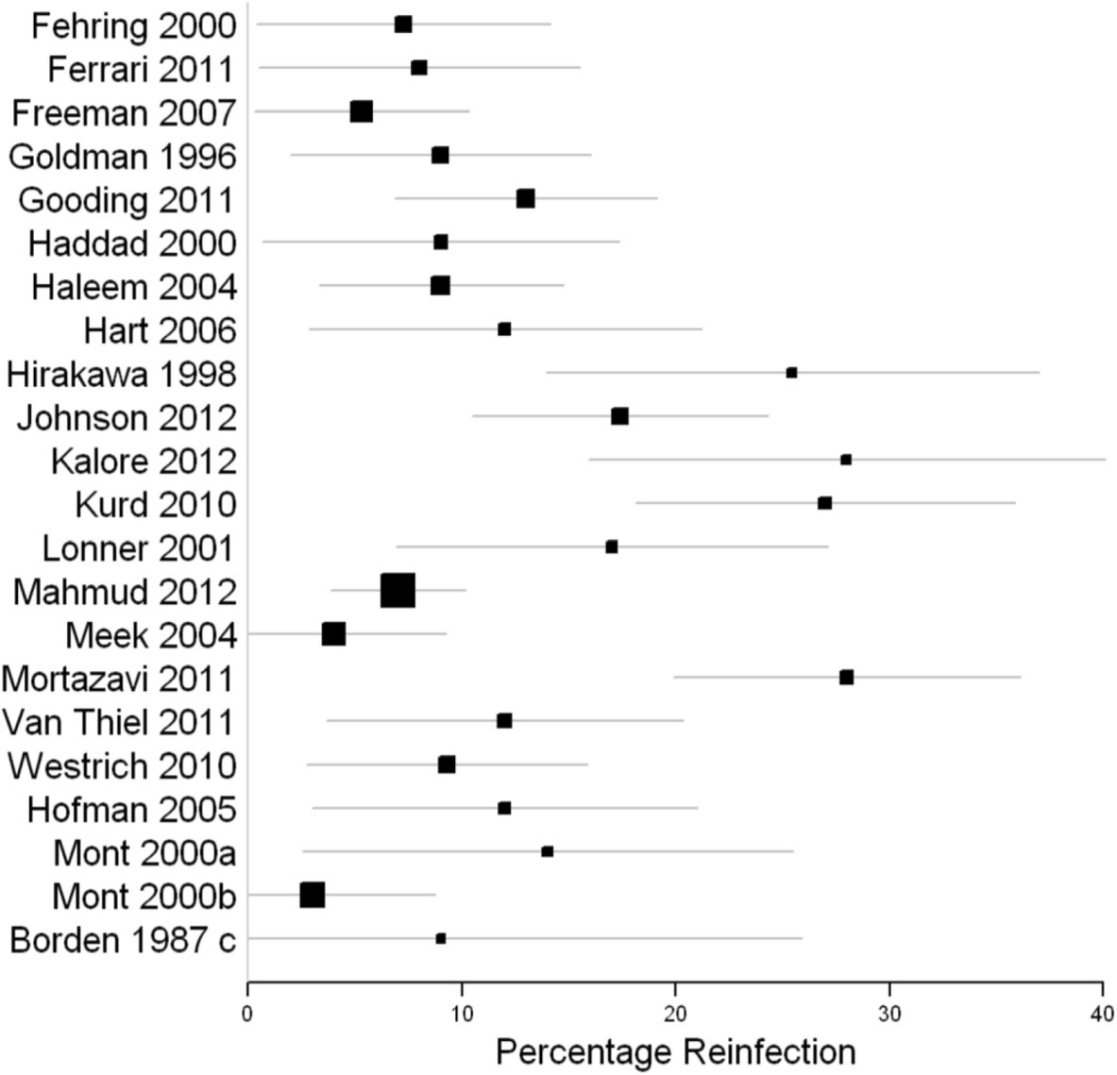
**Forest plot-two stage revisions.** X-axis 1 Point estimate of reinfection rate at last follow-up which is variable (Mont 2000a-group 1 conventional two-stage revision, Mont 2000b-group 2 two stage revision with culture prior to reimplantation) ‘Borden 1987 c’ represents the two stage revision outcome presented in the paper. All estimates are presented with 95% confidence intervals.

58 studies reported the results of two-stage revision for infected total knee arthroplasty [12,24-79]. Studies with greater than 45 knees are summarised in Table 1. The reinfection rates at final follow up are also summarised in Figure 3 presented with 95% confidence intervals. The remaining studies are included in the reference list.

Reinfection rates in all the studies identified range between 0 and 41%. Of the four case series with over 100 patients’ reinfection rates were 7% [51], 13% [35], 17% [44] and 28% [54]. Each of these four studies were published between 2011 and 2012.

The largest study, from Mahmud and colleagues was a retrospective review of 239 patients who underwent two-stage revision for infected TKA. The focus of this study was to establish infection free survivorship at medium interval, which was 85% at 5 years and 78% at 10 years.

They identified 16 patients who were revised for infection, but also identified 17 patients who developed aseptic causes for failure such as loosening, pain and extensor mechanism failure. The authors acknowledge that the diagnosis of periprosthetic infection is not sufficiently robust that they can guarantee these aseptic failures were indeed aseptic [51]. Those patients with successful eradication of infection also demonstrated an improvement in The WOMAC and The Knee Society Clinical Rating scores.

The preoperative WOMAC score, and The Knee Society Clinical Rating scores were 48 and 64, respectively. The postoperative WOMAC and The Knee Society Clinical Rating scores were 60 and 129, respectively.

Johnson et al. (2012) described the treatment of 110 patients (115 knees). Of these, 34 had dynamic spacers and 81 had static spacers. They had comparable rates of re-infection 6/34 (17%) in the dynamic and 14/81 (17%) in the static spacer group. However the authors acknowledge that the groups were unmatched and that the operating surgeon selected spacer type. This study also reports on a number of complications specific to dynamic spacers such as fracture and dislocation of the femoral component.

A similarly sized study from Mortazavi [54] detailed the re-infection rates of 117 knees and sought to identify operative and preoperative risk factors for failure. This group reported a relatively high re-infection rate of 28%. Using multivariate analysis to compare patients who failed two-stage treatment they identified culture negative samples, methicillin resistant organisms and increased re-implantation operative time as predictive of failure. The authors acknowledge that despite the second largest sample size in the literature presently, there is a possibility that the multivariate analysis may be underpowered for some, if not all of the variables under consideration. No functional parameters were reported in this study.

Gooding and colleagues present the outcomes from 115 infected knee replacements [35]. They sought to identify the re-infection rate and the functional outcomes with an articulating, antibiotic impregnated spacer. Fourteen of the 110 patients had a recurrence of infection of which 4 had recurrence with the same organism. 12 of the 14 failures were successfully treated with a further two-stage exchange arthroplasty. The functional arm of this study was limited by fewer than half of the cohort of patients responding to this component of follow up (n = 48). Of these 48 patients the team noted an improvement on SF-12, Oxford and WOMAC functional scores as well as an improvement in flexion deformity. Interestingly the use of logistic regression in this study failed to identify any variables that predicted failure.

An earlier study from 2000 from the same centre of 45 patients demonstrated a 91% infection clearance rate [36], with mean follow up of 48 months (20–112 months). Similarly Haleem and colleagues studied 96 knees in 94 patients with a median follow up of 7.2 years [37]. They reported 9 patients who required implant removal for reinfection. In addition they described a further 6 knees requiring revision for aseptic loosening.

Freeman and colleagues presented the results of 74 patients who underwent 76 two-stage revisions [34]. These patients were further stratified into those who received a static spacer n = 28 and those who received an articulating spacer in the interim period. They had a reinfection rate of 92.1% and 94.7% respectively. Functional comparison of these two groups by way of Knee Society Scores failed to reveal a significant difference in postoperative pain scores or functional scores. However given the limited sample size this may represent a type II error. Similarly there was no significant difference in final range of movement between the groups.

A similarly sized study [65] was the most recent of three studies from one group in the USA. This group’s most recent study of 75 knees in 72 patients had an infection eradication rate of 90.7%. This paper looked at the efficacy of the two-stage technique for antibiotic resistant infections. The eradication rate for resistant organisms was 91.2% (31/34), and 91.3% (42/46) for non-MDR organisms.

Goldman and colleagues report a reinfection rate of 9% in the 64 knees included in their study [12]. Functionally these patients achieved a mean Hospital for special surgery score of 78. Other functional assessments were made using the WOMAC tool, which was carried out in 40 of the studied cohort. 80% of the patients were satisfied with the result of their knee.

Eight studies of between 50 and 60 patients reported reinfection rates of 4% [52], 7% [32], 8% [33], 12% [59] and [70], 25% [40] and 28% [45].

Similarly to the study by Freeman and colleagues, Fehring [32] made a comparison between static (n = 25) and articulating spacers (n = 30). The reinfection rates for the different sub-types of spacer were 12% and 7% respectively. Of interest there was no significant difference in functional scores, and the final average range of movement was 98° in the static group and 105° in the articulating group.

In their cross-sectional study, Meek and colleagues identified a very low re-infection rate for their 54 patients (4%) who underwent two-stage revision with an articulating system [52]. The primary focus of this study was to compare functional outcomes in the septic revisions to those patients who underwent revision for aseptic indications. They found that those who underwent revision surgery for infection fared no worse than those who had surgery for aseptic failure. This was one of the only studies to undertake a power calculation when interpreting their comparison of functional outcomes.

By contrast the studies of Hirakawa [40] and Kalore [45] identified much higher re-infection rates of 25% and 28% respectively. These rates are among the highest of the studies identified by this systematic review.

Hirakawa’s study contained a mixed population of infected knee replacements.

The re-infection rates for two-stage revision of primary TKA was 8%, compared to 41% of the patients who underwent revision of a knee which had undergone multiple previous operations such arthroscopy and osteotomy. This was a significant difference.

### Study design and quality

No study identified in this review used randomisation or blinding in treatment allocation. The overwhelming majority of studies included were single centre case series. The principle limitation of this study design is the lack of a control group for comparison of outcomes. Patient groups are further limited by surgeon selection. This makes generalisation of results difficult to the wider population. In addition a number of the studies are published from large centres; this reduces the external validity of the results.

The wide variation in re-infection rate reported in both one-stage and two-stage procedures may be explained by a number of factors. Firstly the variable definition of re-infection will affect reporting of this complication in the literature. This may be compounded by loss to follow-up and inadequate and absent data collection that frequently complicates retrospective research.

This low methodological quality means that the risk of bias in these studies is very high.

A number of studies undertook a post-hoc analysis of those who failed two-stage revision to identify any of the operative and patient based factors, which may have influenced their failure. Few showed a statistically significant difference, even with risk factors known to influence infection risk such as rheumatoid arthritis. However given the low numbers within each study, a type II error is the most likely explanation.

The forest plots for both one and two stage studies show wide ranging confidence intervals, further emphasising the difficulty in obtaining a true estimate of reinfection for each procedure. Given the gross clinical heterogeneity of the studies, any kind of meta-analysis was deemed to be of low value.

### Summary

This systematic review of exchange arthroplasty for revision of infected knee arthroplasty included 63 original studies. The vast majority of the literature relates to a two-stage protocol (58 studies).

The 9th National Joint Registry of England and Wales report identified that 198 patients had undergone a one-stage revision for infection, compared to 493 patients who had the second of a two-stage revision for infection [1]. This demonstrates that whilst revision for infection might appear to be a marginal topic in the literature, it is a significant problem for practicing surgeons.

Re-infection rates for two-stage procedures varied between 0% and 41% and between 0% and 11% in one-stage procedures. The lower variability in revision rates for the one-stage procedures is likely to reflect the limited number of studies. The large variability between all the studies may also reflect the heterogeneous patients, surgeons and selection criteria. These existing studies are all susceptible to bias.

### Diagnosis of infection

An important controversy in the field of SSI is diagnosis of infection. A number of parameters are usually measured in the serum, knee joint aspirate and tissue samples.

In an effort to provide a universally accepted diagnostic criteria for SSI a work group from the musculoskeletal infection society published a list of criteria based on the current evidence [80]. However this has yet to gain widespread acceptance. Newer techniques are emerging and may help with the current limitations [81,82]. This lack of agreed definition is likely to have significant impact on the re-infection rates reported in the literature identified in our study.

A robust and widely accepted definition of periprosthetic infection will be fundamental to any future studies looking at the efficacy of any interventions for patient with this devastating complication.

### Spacer type

The type of spacer used in two-stage revision was the source of interest in a number of comparative studies identified in this review [32,34,44,56]. Cement spacers that allow the patient to move the knee whilst undergoing antibiotic therapy were introduced to prevent bone loss and soft tissue contraction associated with static spacer use and ultimately patient function [83].

Freeman et al. [34] found significantly more ‘excellent’ knee society scores in patients with dynamic spacers, but no difference in pain scores. Park et al. found their group of patients who received dynamic spacers to have a significantly better range of movement at final follow-up, but no significant difference in functional scoring [56].

Both Johnson [44] and Freeman [34] found no significant difference in functional scores or range of movement between the two groups.

All four of the studies identified comparable infection control between the different spacer types.

### Acceptability to patient

The primary outcome measure of this systematic review is the re-infection rate. Whilst an undoubtedly important outcome in revision for infected knee arthroplasty, it is unable to describe patient satisfaction. With an ever increasing emphasis on patient reported outcomes and patient expectation any future work must take into account the acceptability to patients. Some specific work on functional outcomes in patients who have undergone two-stage revision identifies that even those who successfully cleared infection failed to return to vigorous activity [84]. A retrospective parallel case series looked at Oxford Knee score, EQ5D and satisfaction in patients who had undergone single stage and two stage revision [85]. They showed patients in each group to have similar outcomes in all measures used. The authors recommended decisions about technique should be based upon re-infection rate.

### Economics

Kurtz and colleagues investigated the cost burden associated with the SSI [2]. They identified that knee arthroplasty associated with infection was associated with significantly longer inpatient stay and hospital charges. Whilst no sub-analysis was undertaken as to the different costs associated with one and two stage revision, there is likely an economic benefit to be had if one-stage revision can eradicate infection as reproducibly as two-stage revision.

### Limitations

Limitations of this study relate to the narrative presentation of results that are necessarily selective. This is potentially a source of dispute and disagreement. Furthermore this study encompassed only English language studies, which was a practical limitation. We note that in previous reviews[14,86] that there has been reference to a number of studies on single stage revision published in German [87] French[88] and Chinese [89].

### Risk factors for failure and directions for future practice

Intuitively risk factors for failure of primary arthroplasty ought to remain true for revision arthroplasty. However this is less well proven in the literature. The review by Siva and colleagues in 2002 identifed gram-positive organisms, the absence of sinus formation, use of antibiotic-impregnated bone cement for fixation of the new prosthesis, and long-term use of antibiotic therapy as being associated with successful single stage revision [90]. A review of revision hip surgery alluded to similar risk factors for predicting success of single stage revision [91].

The study from Mortazavi and colleagues sought to identify risk factors for failure of two stage procedures. They identified ‘culture negative’ infection, methicillin resistant organism and extended reimplantation operative time were significantly associated with failure in their cohort [54].

Selecting patients for revision surgery should consider these factors. This has been recognized by proponents of single stage revision who advocate its use in immunocompetent patients with a known organism, in the absence of osteitis, sinus and bone loss [14,15].

## Conclusion

The perceived gold standard for revision of infected knee arthroplasty is the two-stage procedure. The studies identified in this systematic review demonstrate a much larger body of evidence to support the use of this technique over a single stage procedure. However none of the studies described here offer definitive evidence to support either technique.

Given the clear benefits for patients who befall this complication, if non-inferiority were demonstrated for the one-stage technique then one would expect the technique to gain wider acceptance. This would be best achieved in a large scale multicentre prospective randomised clinical trial.

## Competing interests

The authors declare that they have no competing interests.

## Authors’ contributions

JM and NS designed the search strategy, conducted the search and collected the studies. Where discrepancies arose agreement was reached by discussion. JM drafted the manuscript with NS. HP did the statistical analyses and produced the forest plots. MR and PF provided critical revisions to the pre-submission manuscript. AS conceived of the study, supporting JM and NS throughout the conduct of the review process. AS also provided important critical revisions. All authors read and approved the final manuscript.

## Pre-publication history

The pre-publication history for this paper can be accessed here:

http://www.biomedcentral.com/1471-2474/14/222/prepub

## Supplementary Material

Additional file 1: Table S1 Search strategy used in Embase. **Table S2.** Search strategy used in MEDLINE. Click here for file

Additional file 2 Excluded studies. Click here for file
