# Supplementary material for: A systematic review of the evidence for single stage and two stage revision of infected knee replacement
Source: BMC Musculoskelet Disord. 2013 Jul 29;14:222. doi: 10.1186/1471-2474-14-222 (PMC3734185; doi:10.1186/1471-2474-14-222)
Supplement: Additional file 1: Table S1 — Search strategy used in Embase. Table S2. Search strategy used in MEDLINE. [file 1471-2474-14-222-S1.docx]

Additional File

Table 1 Search strategy used in Embase

| 1 | prosthesis-related infections.mp. or infection/ |
| --- | --- |
| 2 | exp wound infection/ |
| 3 | surgical infection/ |
| 4 | infect*.mp. or infection/ |
| 5 | arthroplasty, replacement, knee.mp. or exp knee arthroplasty/ |
| 6 | exp knee prosthesis/ |
| 7 | knee arthroplasty/ |
| 8 | knee replacement.mp. |
| 9 | total knee.tw. |
| 10 | 2-stage.tw. |
| 11 | two stage.tw. |
| 12 | 1-stage.tw. |
| 13 | one stage.tw. |
| 14 | exchange.mp. |
| 15 | 1 or 2 or 3 or 4 |
| 16 | 5 or 6 or 7 or 8 or 9 |
| 17 | 10 or 11 or 12 or 13 or 14 |
| 18 | 15 and 16 and 17 |

Table 2-Search strategy used in MEDLINE

| 1 | exp Sepsis/ or exp Prosthesis-Related Infections/ or exp Surgical Wound Infection/ |
| --- | --- |
| 2 | exp Infection/ or exp Wound Infection/ |
| 3 | infect*.mp. |
| 4 | 1 or 2 or 3 |
| 5 | exp Arthroplasty, Replacement, Knee/ or exp Knee Prosthesis/ |
| 6 | total knee.mp. |
| 7 | TKR.mp. |
| 8 | (knee replacement or knee arthroplasty or knee revis*).mp. |
| 9 | 5 or 6 or 7 or 8 |
| 10 | (1-stage or one stage).mp. |
| 11 | (2-stage* or two stage*).mp. |
| 12 | exchange*.mp. |
| 13 | 10 or 11 or 12 |
| 14 | 4 and 9 and 13 |
