# Supplementary material for: A systematic review of the evidence for single stage and two stage revision of infected knee replacement
Source: BMC Musculoskelet Disord. 2013 Jul 29;14:222. doi: 10.1186/1471-2474-14-222 (PMC3734185; doi:10.1186/1471-2474-14-222)
Supplement: Additional file 2 — Excluded studies. [file 1471-2474-14-222-S2.doc]

Additional file 1

**Excluded studies**

| **Study** | **Reason for exclusion** | |
| --- | --- | --- |
| Ascione 2011 | Excl-Only used 2 stage in subgroup/no separate reporting of hip and knee data | |
| Bengtson 1989 | Excl-No reporting of individual outcome of two stage/mixed types of knee | |
| Bengtson 1991 | Excl-One stage and two stage not reported separately | |
| Berbari 2006 | Excl-No joint specific information available | |
| Berbari 2007 | Excl-No joint specific information available | |
| Bliss 1985 | Excl- Insufficient follow up | |
| Booth 1989 | Excl- Insufficient follow up | |
| Buechel 2004 | Excl-Review |  |
| Chiang 2011 | Excl-Subgroup of resistant microorganisms | |
| Cismaiu 2011 | Excl-no seperate reporting of hip/knee data | |
| Cordero-Ampuero 2010 | Excl-Subgroup of resistant microorganisms | |
| Estes 2010 | Excl-Not 2 stage revision | |
| Gardner 2011 | Excl-Not a series of 2 stage revision | |
| Hoad-Reddick 2005 | Excl-Mixed treatment types unable to elucidate failure in completed two stage revision |  |
| Hsu 2008 | Excl- Insufficient follow up | |
| Jamsen 2006 | Excl- Insufficient follow up | |
| Jamsen 2009 | Excl-systematic review | |
| Kim 2011 | Excl-Mixed treatment types/patients stratified | |
| Kosters 2009 | Excl-Follow up too short | |
| Kubista 2012 | Exl-Insufficient follow up | |
| Laffer 2006 | Excl-Insufficient follow up | |
| Macmull 2010 | Excl-Mixed population of implants-principally revision of revision implants | |
| Masri 1994 | Excl Insufficient follow-up | |
| McPherson 1997 | Excl-Not a simple 2 stage revision-use of gastroc flap | |
| Michalak 2006 | Excl-Subgroup bone loss and use of allograft | |
| Mittal 2007 | Excl-Subgroup of resistant microorganisms | |
| Nickinson 2012 | Excl-Subgroup of bone loss | |
| Parvizi 2009 | Excl-Mixed treatment types | |
| Qiu 2010 | Excl-Selected group of medical co-morbidities | |
| Rasouli 2012 | Excl-Subgroup of specific microorganisms | |
| Ritter 2010 | Excl-Insufficient follow up | |
| Siebel 2002 | Excl-Native infected joints under consideration | |
| Siegel 2001 | Excl-Review |  |
| Silva 2002 | Excl-Only used 2 stage in subgroup/too few in number | |
| Teeny 1990 | Excl-Not a series of 2 stage revision | |
| Tintle 2009 | Excl-Irrigation and exchange | |
| Van Kleunen 2010 | Excl; Prosthesis retention |  |
| Windsor 1990 | Excl-Subgroup of specific microorganism | |
| Zmistowski 2011 | Excl-Selected group of medical co-morbidities | |
| Emerson 2002 | Excl No specific description of revision procedure | |
| Wang 2004 | Excl-fewer than 5 patients | 1 |
| Grogan 1986 | Excl Insufficient follow up | |
| Morrey 1989 | Excl-Review |  |
| Nelson 2001 | Excl-Review |  |
| Brause 1982 | Excl-Review |  |
